# Systematic Review and Meta-Analysis: Can We Compare Direct Oral Anticoagulants to Warfarin in Patients With Atrial Fibrillation and Bio-Prosthetic Valves?

**DOI:** 10.7759/cureus.14651

**Published:** 2021-04-23

**Authors:** Govinda Adhikari, Nischit Baral, Rohit Rauniyar, Sandip Karki, Basel Abdelazeem, Pramod Savarapu, Sakiru Isa, Hafiz Muhammad Waqas Khan, Mahin R Khan, Hameem U Changezi

**Affiliations:** 1 Internal Medicine, McLaren Flint, Flint, USA; 2 Cardiology, McLaren Flint, Flint, USA

**Keywords:** direct oral anticoagulant therapy, bio-prosthetic valve, major bleeding events, intracranial hemorrhage, atrial fibrillation, stroke occurrence, systematic review and meta-analysis

## Abstract

Background

There are no clear consensus guidelines on the indications and types of anticoagulation therapies in patients with bio-prosthetic valves either with concomitant atrial fibrillation (AF) or sinus rhythm. In our meta-analysis, we assessed the safety and efficacy of DOACs as compared to the standard treatment with warfarin in patients with AF and bioprosthetic valves.

Methods

We included randomized controlled trials (RCTs), cohort studies in the English language, and studies reporting patients with valvular heart disease that included bioprosthetic valvular disease. A systematic literature review using Embase, PubMed, and Web of Science was performed using the terms “Direct Acting Oral Anticoagulant,” “Oral Anticoagulants,” “Non-Vitamin K Antagonist Oral Anticoagulant,” “Atrial Fibrillation,” “Bioprosthetic Valve” for literature published prior to January 2021. Extraction of data from included studies was carried out independently by three reviewers from Covidence. We assessed the methodical rigor of the included studies using the modified Downs and Black checklist.

Results

Four RCTs and one observational study (n=1776) were included in our study. A random-effect model using RevMan (version 5.4; The Nordic Cochrane Centre, The Cochrane Collaboration, Copenhagen) was used for data analysis. The pooled data showed that there was a non-significant reduction in the incidence of stroke and systemic embolism in the patients taking DOACs as compared to warfarin (HR 0.69; 95% CI, 0.29, 1.67; I^2 ^= 50%). The incidence of major bleeding was lower in the DOACs group; the difference was statistically significant (HR 0.42; 95% CI, 0.26, 0.67; I^2^ = 7%). The difference was not statistically significant for all-cause mortality in both groups (HR 1.24; 95% CI, 0.91, 1.67; I^2^ = 0%).

Conclusion

Our results showed that there was no difference in the outcomes of stroke and systemic embolism between DOACs and warfarin but there were statistically significantly lower major bleeding events. We conclude that larger clinical trials are needed to assess the true safety and efficacy of DOACs in patients with AF and bioprosthetic valves.

## Introduction

Direct oral anticoagulants (DOACs) are a newer group of anticoagulants that inhibit Factor Xa (rivaroxaban, apixaban, edoxaban, betrixaban) or Factor IIa/thrombin (dabigatran). Vitamin K antagonists (VKAs) have been the choice for thromboembolic prophylaxis and treatment for many years [1]. However, DOACs emerged as the most frequently used anticoagulants in practice to reduce stroke and systemic embolism. In contrast to VKAs, DOACs have immediate onset and offset of action and do not require frequent monitoring [2]. On the other hand, VKAs have food and drug interactions, a narrow therapeutic window, and genetic susceptibility imposing challenges to clinicians for frequent dose adjustments to achieve the target therapeutic internationalized normalized ratio [3].

American Heart Association (AHA)/American College of Cardiology (ACC)/Heart Rhythm Society (HRS) (2019) guidelines for the management of patients with atrial fibrillation (AF) define non-valvular AF as AF in the absence of moderate-to-severe mitral stenosis or a mechanical heart valve [4]. However, these terminologies, valvular and non-valvular AF, have been confusing due to differences in different society guidelines and variations in definitions used in different randomized controlled trials. The European Society of Cardiology recommends avoiding the use of such terminologies [4-5]. AHA/ACC/HRS (2019) guidelines recommend DOACs over warfarin in eligible patients with atrial fibrillation except for moderate-to-severe mitral stenosis or a mechanical heart valve [4-5]. However, a clear consensus guideline on anticoagulation in patients with atrial fibrillation and bioprosthetic valve is lacking. We attempted to do a metanalysis of four randomized controlled trials and one observational study comparing the safety and efficacy of DOACs versus warfarin in patients with atrial fibrillation and a bioprosthetic valve.

## Materials and methods

Search strategy

The Preferred Reporting Items for Systematic Reviews and Meta-Analyses (PRISMA) statement for reporting systematic reviews as recommended by the Cochrane Collaboration was followed in this systematic review. An electronic database using Embase, PubMed, and Web of Science was performed using the terms “Direct Acting Oral Anticoagulant,” “Oral Anticoagulants,” “Non-Vitamin K Antagonist Oral Anticoagulant,” “Atrial Fibrillation,” and “Bioprosthetic Valve” for literature published prior to January 2021.

Inclusion and exclusion criteria

We included randomized controlled trials (RCTs) and cohort studies in the English language; studies reporting patients with valvular heart disease that included bioprosthetic valvular disease. We included all studies that compared DOACs with warfarin in AF that have clearly reported the presence of bioprosthetic valve and reported events of stroke, systemic embolism, all-cause mortality, and major bleeding. We excluded studies involving patients with a valvular disease requiring surgery, hemodynamically unstable patients with valvular disease, mechanical heart valves, rheumatic valvular disease, including moderate to severe mitral stenosis. In addition, studies that did not report stroke, systemic embolism, and major bleeding outcomes separately were also excluded.

Data collection and processing

Search results were saved in EndNote files and transferred into Covidence. Two reviewers (GA and RR) independently performed the title and abstract screening and full-text screening. Conflicts were resolved through consensus. Extraction of data from included studies was carried out independently by three reviewers (GA, NB, RR) from Covidence.

Methodical quality assessment

We assessed the methodical rigor of the included studies using the modified Downs and Black checklist for RCTs and non-randomized studies. The checklist has 27 items with a total possible score of 28. Papers were rated excellent if they scored above 25, good if they scored between 20 and 25, fair if they scored between 15 and 19, and poor if they scored <15. Each study was assessed by two independent investigators and discrepancies in scoring were resolved using consensus. The risk of bias across studies was not assessed because of fewer studies in our meta-analysis.

Measure of outcome

The primary efficacy outcome of interest was composite of stroke (ischemic, hemorrhagic, and undetermined stroke) and systemic embolism. The primary safety outcome was major bleeding in accordance with the definition from the International Society of Thrombosis and Hemostasis (ISTH) guidelines while the secondary safety outcomes were intracranial hemorrhage (ICH) and all-cause mortality.

Statistical analysis

Outcomes from the individual studies were aggregated with RevMan (version 5.4, Cochrane Collaboration, Oxford, United Kingdom) applying the Mantel-Haenszel test. Hazard ratio (HR) and 95% confidence intervals (CIs) were estimated using a random-effects method to account for the presence of variability among the studies. The I2 statistic was used to assess heterogeneity. Two-tailed p-values <.05 were considered to indicate statistical significance.

Study selection

**Figure 1:**
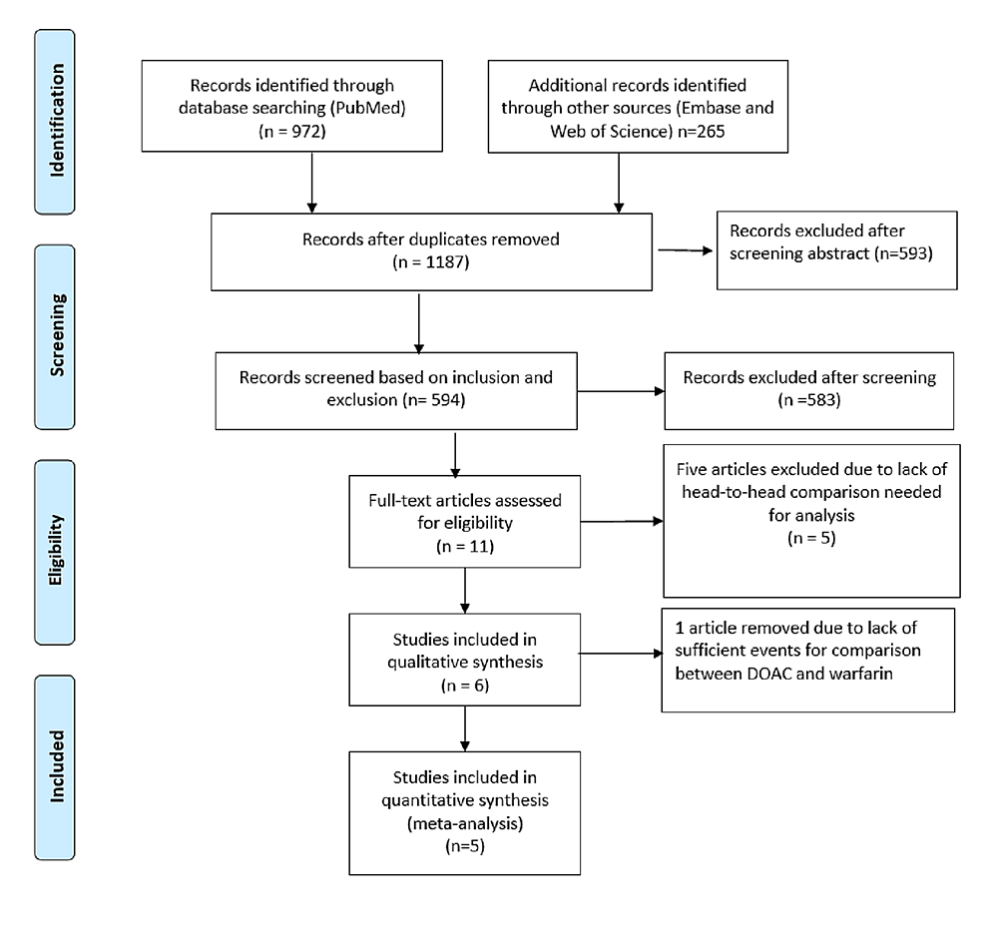
PRISMA diagram of included studies DOAC: Direct Oral Anticoagulant

From PubMed, we identified 972 articles, and from Embase 262 and Web of Science, we identified three articles. The duplicated studies were removed by the software. When we analyzed the remaining 594 studies based on inclusion and exclusion criteria, we identified 11 studies. Finally, 11 articles were fully read and five articles removed. Final qualitative and quantitative analysis was done with four studies (Figure 1).

Baseline characteristics of included studies

We included four studies for meta-analysis and one study for reference (Table 1).

**Table 1:** Baseline characteristics of studies included in our meta-analysis NA: not available; SE: systemic embolism; AF: atrial fibrillation

Author	Study type	Study design	Country	Study/Patient characteristics	Intervention	Participants	Age	CHA2DS2-Vasc	HAS-BLED	Previous major bleeding	Previous stroke/SE
Strange et al. [6]	Subgroup analysis	Observational, prospective cohort study	Denmark	Nationwide registry in patients with AF and aortic stenosis/insufficiency, mitral insufficiency, bioprosthetic valves, mitral and aortic valve repair	Rivaroxaban and Apixaban; Warfarin	180; 217	NA	NA	NA	NA	NA
Guimarães et al. [7]	Subgroup analysis (Aristotle Trial)	Randomized, double-blind controlled trial	Multinational (41 nations)	Males and females ≥ 18 yrs with atrial fibrillation (AF) and one or more of the following risk factors for stroke: Age ≥ 75, previous stroke, transient ischemic attack (TIA) or systemic embolism (SE), symptomatic congestive heart failure, or left ventricular dysfunction with left ventricular ejection fraction (LVEF) ≤ 40%, diabetes mellitus, or hypertension requiring pharmacological treatment.	Apixaban; Warfarin	87; 69	72; 74	0-1 (31); 2 (26) ≥3 (30); 0-1 (18); 2 (28) ≥3 (23)	0-1 (24) 2; (32) ≥3 (31); 0-1 (18); 2 (28) ≥ 3 (23)	25; 19	24; 12
Durães et al. [8]	Original research article (DAWA Trial)	Phase II, prospective, randomized open-labeled trial	Brazil	Patients with mitral and/or aortic bioprosthesis valve replacement at least three months prior to entering the study and had documented AF postoperatively.	Dabigatran; Warfarin	15; 12	48.8; 45.7	NA	0; 0	0; 0	4; 4
Guimarães et al. [9]	Original Research Article (RIVER Trial)	Randomized controlled trial open-label design	Brazil	Patients with age more than 18 years with AF and a bio-prosthetic valve only were included, excluding all other valvular lesions and mechanical valves.	Rivaroxaban; Warfarin	500; 505	59.4; 59.2	2.7; 2.5	1.6; 1.6	NA; NA	63; 66
Carnicelli et al. [10]	Subgroup analysis from ENGAGE AFTIMI 48	Randomized double-blind controlled trial	Multinational study	Patients with age more than 21 and CHADS_2 _score 2 with a history of documented AF within 1 year	Edoxaban; Warfarin	121; 70	NA	NA	NA	NA	NA

## Results

Quality of included studies

We used the Downs and Black tool for assessing the quality of included studies, which showed that four of our studies had an excellent quality and one of the studies was only a good study (Table 2). 

**Table 2:** Downs and Black tool for quality of included studies E: Excellent; G: Good; Q: Quality of Study; R: Reporting; EV: External Validity; IV: Internal Validity; C: Confounder; P: Power; T: Total Score

STUDY ID	R										E V		IV						C								P	T	Q
Question No.	1	2	3	4	5	6	7	8	9	10	11	12	13	14	15	16	17	18	19	20	21	22	23	24	25	26	27		
Guimarães 2020 [9]	1	1	1	1	2	1	1	1	1	1	1	1	1	0	1	1	1	1	1	1	1	1	1	0	1	1	1	26	E
Guimarães 2019 [7]	1	1	1	1	2	1	1	1	1	1	1	1	0	1	1	1	1	1	1	1	1	1	1	1	1	1	1	27	E
Strange 2020 [6]	1	1	1	1	2	1	1	1	0	1	0	1	1	0	0	1	1	1	1	1	1	1	0	0	0	0	1	20	G
Carnicelli 2017 [10]	1	1	1	1	2	1	1	1	1	1	1	1	0	1	1	1	1	1	1	1	1	1	1	1	1	1	1	27	E
Durães 2016 [8]	1	1	1	1	2	1	1	1	1	1	1	1	1	0	1	1	1	1	1	1	1	1	1	0	1	1	1	26	E

Stroke and systemic embolism

**Figure 2:**
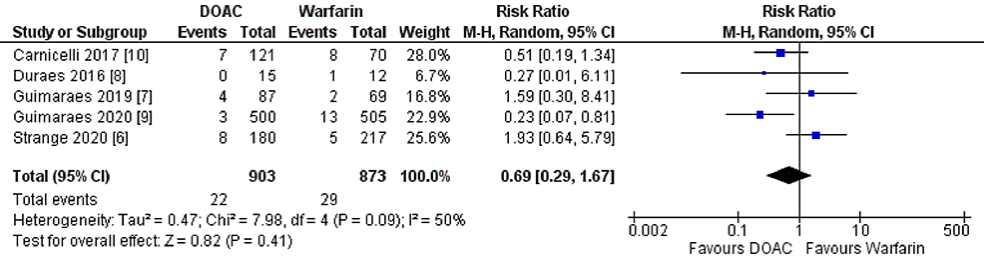
Forest plot showing a comparison of stroke/systemic embolic events between DOAC and Warfarin DOAC: Direct Oral Anticoagulant

The pooled data from five studies showed stroke and systemic embolism in 22 out of 903 (2.44%) patients in the DOAC group and 29 out of 873 (3.32%) patients in the warfarin group. The pooled result showed that there was a non-significant reduction in the incidence of stroke and systemic embolism in the patients taking DOACs compared to warfarin (HR 0.69; 95% CI, 0.29, 1.67; heterogeneity I2 = 50%) (Figure 2).

Major bleeding

**Figure 3:**
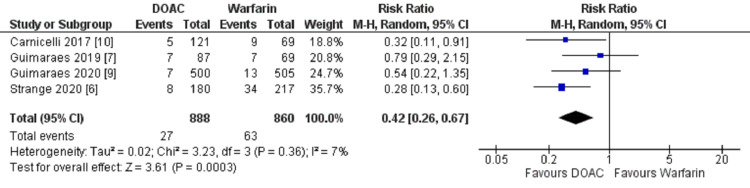
Forest plot showing a comparison of major bleeding between DOAC and Warfarin. DOAC: Direct Oral Anticoagulant

The pooled data from four studies showed major bleeding in 27 out of 888 (3.04%) in the DOACs group and 63 of 860 (7.32%) patients in the warfarin group. The pooled result showed that the incidence of major bleeding was lower in the DOAC group as compared to warfarin; the difference was statistically significant. (HR 0.42; 95% CI, 0.26, 0.67; heterogeneity I2 = 7%) (Figure 3).

Intracranial hemorrhage

**Figure 4:**
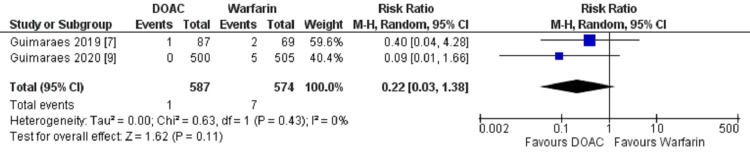
Forest plot showing a comparison of ICH events between DOAC and Warfarin. DOAC: Direct Oral Anticoagulant; ICH: Intracranial Hemorrhage

The pooled data from two studies that reported the events of intracranial hemorrhage (ICH) in one out of 587 (0.17%) in the DOACs group as compared to seven out of 574 (1.21%) in the warfarin group. Composing the data from two studies, the pooled result showed that there was a non-significant reduction in the incidence of Intracranial hemorrhage in the patients taking DOACs versus warfarin (HR 0.22; 95% CI, 0.03, 1.38; heterogeneity I2 = 0%) (Figure 4).

All-cause mortality

**Figure 5:**
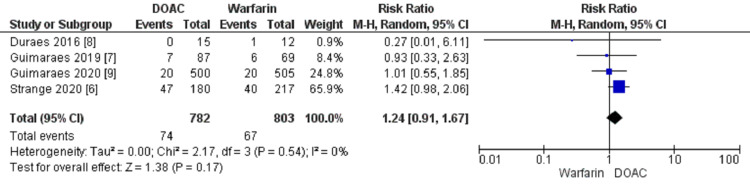
Forest plot showing a comparison of all-cause mortality between DOAC and warfarin DOAC: Direct Oral Anticoagulant

The pooled data from four studies showed all-cause mortality in 74 out of 782 (9.46%) patients in the DOACs group and 67 out of 803 (8.34%) patients in the warfarin group. The pooled result showed that although the incidence of all-cause mortality was higher in the DOAC group as compared to warfarin; the difference was not statistically significant (HR 1.24; 95% CI, 0.91, 1.67; heterogeneity I2 = 0%) (Figure 5). 

## Discussion

Bioprosthetic valves are indicated in the elderly ≥ 65 years old, in patients with limited life expectancy, women of childbearing age who desire to be pregnant, and in those where anticoagulation is not an option [11]. The hypercoagulability of pregnancy further increases the thromboembolic risk in those with mechanical valves [12]. There is an increasing incidence of atrial fibrillation and heart valve disease with age. The incidence of atrial fibrillation is estimated to rise to 2.6 million cases and prevalence to 12.1 million cases by 2030 [13]. Also, there is a growing trend toward the use of bioprosthetics for heart valve disease repair [14-15].

Patients with mechanical valves have lower re-operation rates but higher thromboembolic risk compared to bioprosthetic valves and indefinite anticoagulation with VKAs is recommended [11,16]. The introduction of results of the RE-ALIGN trial discouraged the use of novel oral anticoagulants (NOACs) in mechanical prosthetic valves. European Medicines Agency’s Committee for Medicinal Products for Human added contraindication to Pradaxa (dabigatran) due to increased thrombotic and bleeding events seen in the trial [17-18]. Although rare, those with bioprosthetic valves are also at increased risk for valve thrombosis and systemic embolization [11,16]. The risk is heightened during the early postoperative period during endothelialization of the suture zone [16]. Hence, considering anticoagulation in patients with bioprosthetic valve repair during the early postop period should be reasonable. However, there are no clear consensus guidelines on indications and type of anticoagulation therapy in patients with bioprosthetic valve either with concomitant atrial fibrillation or sinus rhythm.

Our metanalysis did not show any significant difference in the incidence of stroke and systemic embolism in patients with bioprosthetic valves taking DOACs compared to warfarin for atrial fibrillation. However, the major bleeding events were significantly lower in the DOACs group. In a metanalysis by Caldeira, et al. (2017), secondary analysis of bioprosthetic valves depicted that the thromboembolic complications and major bleeding were similar in both groups. However, NOACs significantly reduced stroke and systemic embolism in both valvular and non-valvular heart disease groups compared to warfarin with no significant difference in major bleeding risk [17]. The study had excluded mechanical and rheumatic mitral valvular atrial fibrillation in the valvular heart disease group. One of the propensities-matched retrospective cohort studies (n=24) showed no events of stroke or systemic embolism in either NOAC or warfarin in patients with bioprosthetic valves [19]. In a small (n=73), single-center retrospective cohort study of NOACs in the bioprosthetic valve, there was one transient ischemic attack (TIA) event and 6.9% had major bleeding events [20]. However, the majority of patients were on concomitant aspirin therapy, which might be the confounding factor for the observed result.

There was also no significant difference in all-cause mortality or intracranial hemorrhage in our study. Only two studies were analyzed for intracranial hemorrhage because it was not reported in other studies. However, DOACs significantly reduced intracranial hemorrhage in patients with both valvular and non-valvular heart disease compared to warfarin in the study by Caldeira et al. (2017). 

Similar results were evident in studying RCTs alone in our study. The recent meta-analysis by Kheiri B et. al. involving RCTs alone showed results similar to our study in all outcomes except major bleeding [21]. The major bleeding event was statistically non-significant in their study. We used the combined major bleeding events of low and high-dose edoxaban from the RCT by Carnicelli et al. for data synthesis in our meta-analysis. We calculated the number needed to harm as 55.55.

Limitations

Our study included a meta-analysis of different DOACs, which can add to the heterogeneity of pooled results. All studies did not include all the secondary safety outcomes like ICH and major bleeding. Also, the sample size was small in our study. Moreover, we included observational studies comparing DOAC with warfarin. The lack of blinding and allocation concealment in these studies can cause biases in the observed results.

## Conclusions

With increasing incidences of atrial fibrillation and heart valve disease with age and increasing trends in bioprosthetic valve replacement, it is likely for a clinician to encounter an increasing number of patients with bioprosthetic valve and atrial fibrillation. Our study shows that DOACs have comparable efficacy with warfarin with statistically significantly lower bleeding events. However, with the various study limitations discussed above, there is a need for focused studies and larger trials to guide the safer anticoagulation option in such a group of patients.
